# Association of Smoking, Alcohol Use, and Betel Quid Chewing with Epigenetic Aberrations in Cancers

**DOI:** 10.3390/ijms18061210

**Published:** 2017-06-06

**Authors:** Tong-Hong Wang, Shih-Min Hsia, Yin-Hwa Shih, Tzong-Ming Shieh

**Affiliations:** 1 Tissue Bank, Chang Gung Memorial Hospital, Taoyuan 33305, Taiwan; cellww@gmail.com; 2 Research Center for Industry of Human Ecology, Chang Gung University of Science and Technology, Taoyuan 33303, Taiwan; 3 Graduate Institute of Health Industry Technology, Chang Gung University of Science and Technology, Taoyuan 33303, Taiwan; 4 Liver Research Center, Chang Gung Memorial Hospital, Taoyuan 33305, Taiwan; 5 School of Nutrition and Health Sciences, College of Nutrition, Taipei Medical University, Taipei 11031, Taiwan; Bryanhsia@tmu.edu.tw; 6 Nutrition Research Center, Taipei Medical University Hospital, Taipei 11031, Taiwan; 7 Department of Oral Hygiene, College of Dental Medicine, Kaohsiung Medical University, Kaohsiung 80708, Taiwan; 8 Department of Dental Hygiene, College of Health Care, China Medical University, Taichung 40402, Taiwan

**Keywords:** smoking, alcohol, betel quid, cancer, epigenetics

## Abstract

Numerous environmental factors such as diet, alcohol use, stress, and environmental chemicals are known to elicit epigenetic changes, leading to increased rates of cancers and other diseases. The incidence of head and neck cancer, one of the most common cancers in Taiwanese males, is increasing: oral cancer and nasopharyngeal carcinoma are ranked fourth and tenth respectively, among the top ten cancers in this group, and a major cause of cancer-related deaths in Taiwanese males. Previous studies have identified smoking, alcohol use, and betel quid chewing as the three major causes of head and neck cancers; these three social habits are commonly observed in Taiwanese males, resulting in an increasing morbidity rate of head and neck cancers in this population. In this literature review, we discuss the association between specific components of betel quid, alcohol, and tobacco, and the occurrence of head and neck cancers, lung cancer, gastrointestinal cancers, and urethral cancer. We focus on regulatory mechanisms at the epigenetic level and their oncogenic effects. The review further discusses the application of FDA-approved epigenetic drugs as therapeutic strategies against cancer.

## 1. Introduction

Inappropriate diet, alcoholism, or exposure to toxic chemicals are all known to increase the risk of disease [1,2]. It has been shown that this increased risk may be attributed to, among other factors, epigenetic changes that result in abnormal cell physiology [3,4,5,6]. Epigenetic modification is defined as chromosomal modifications that result in changes to gene expression without alteration of the DNA sequence. Classical epigenetic regulation involves DNA methylation and histone modification, in which modification of DNA or histones, respectively, alters chromatin structure, thereby affecting gene expression. Epigenetic modifications in various species are known to regulate gene expression in a spatiotemporal manner in the physiological context as well as during development. Epigenetic regulation plays a critical role during embryonic development as well as in disease; dysfunctional epigenetic regulation, which results in aberrant gene expression, is closely linked with various diseases such as cancer [7,8,9]. In addition to endogenous intracellular factors, various environmental factors such as diet, environmental chemicals, or exposure to stress are known to affect epigenetic processes, thereby increasing the risk of disease [10]. In addition, numerous reports have shown that the exposure of pregnant women to harmful environmental factors results in epigenetic abnormalities in the embryo such as growth retardation [11,12,13].

Betel quid chewing, smoking, and alcohol use are the three major risk factors for oral cancer and throat cancer [14,15]. In Taiwan, ~2 million people chew betel quid recreationally; among these, 90% are reported to smoke tobacco and drink alcohol simultaneously [14,16]. Several recent reports have shown that betel quid chewing combined with smoking or drinking significantly increases the risk of oral and throat cancers. These effects are attributed to the presence of carcinogens, such as arecoline and arecaidine, in betel quid and alcohol. Such carcinogens exert oncogenic effects via long-term stimulation of, and the induction of epigenetic changes in, oral mucosal cells, resulting in the aberrant expression of oncogenes and tumor suppressors such as *p53*, *BRCA2*, and *XRCC4* [17,18,19,20,21]. This literature review focuses on three carcinogenic factors (betel quid chewing, smoking, and alcohol) and their role in eliciting epigenetic changes associated with head and neck cancers, gastrointestinal cancers, and other malignant tumor types.

## 2. Epigenetic Modification of DNA

### 2.1. DNA Methylation

Methylation of DNA is defined as the addition of a methyl group from the methyl donor *S*-adenosylmethionine (SAM) to a cytosine base within the DNA. This reaction is catalyzed by families of DNA methyltransferases (DNMTs), including DNMT1, DNMT2, and DNMT3 [22]. DNA methylation most commonly occurs on CpG dinucleotides; however, several instances of non-CpG methylation have been found to occur in mammals [23,24]. Most CpG dinucleotide methylation occurs outside of CpG-rich clusters, which also known as CpG islands; these are defined as 500–3000-bp DNA segments that exhibit at least 50% CG content and a ratio of observed CpGs to expected CpGs of greater than 0.6 [25,26,27]. There are approximately 50,000 CGIs in the human genome, several of which have been found in or near approximately 40% of the promoters of mammalian genes [28,29,30]. DNA methylation inhibits the binding of transcription factors directly by “masking the DNA” or indirectly by recruiting methyl-CpG binding proteins (MBPs), which possess repressive chromatin-remodeling activities [31,32].

### 2.2. Epigenetic Modification of Histones

#### 2.2.1. Histone Modification

Chromatin is composed of nucleosomes, which comprise 146 bp of DNA wrapped around an octamer containing two copies of four histone proteins (H2A, H2B, H3, and H4). Histones are basic proteins that regulate the compaction of chromatin and modulate gene expression by altering chromatin structure. Most histone modifications occur at their unstructured alkaline N-terminal tails, via acetylation, methylation, phosphorylation, ubiquitination, and SUMOylation [33,34,35]. These post-transcriptional modifications collectively regulate chromatin structure, which affects biological processes such as gene expression, DNA repair, and chromosome condensation.

#### 2.2.2. Acetylation

Histone acetylation, which most commonly occurs at basic amino acids lysine and arginine, is the most widely studied epigenetic protein modification [36]. Histone acetylation is mediated by histone acetyltransferases (HATs) and histone deacetylases (HDACs). Activation of gene expression by HATs involves the acetylation of specific lysine residues in histone tails, resulting in the removal of the positive charge on histones. This reduces the affinity of histones for DNA, thereby relaxing DNA structure and enhancing transcription. This effect may be reversed by HDAC activity, resulting in transcription suppression.

#### 2.2.3. Methylation

Histone methylation occurs on both lysine and arginine residues. The most extensively studied histone methylation sites include histone H3 lysine 4 (H3K4), H3K9, H3K27, H3K36, H3K79, and H4K20. Sites of arginine methylation include H3R2, H3R8, H3R17, H3R26, and H4R3 [37]. However, methylation at numerous other basic residues present on histone proteins H1, H2A, H2B, H3, and H4 has also been recently shown to occur [38]. Histone methylation is controlled by histone methyltransferases (HMTs) specific to lysine and arginine residues, as well as by histone demethylases (HDMs) such as peptidylarginine deiminase (PADI), Jumonji-C domain-containing histone demethylases (JMJCs), and lysine-specific demethylases (LSDs) [32]. Histone methylation causes transcription repression or activation, depending on the target sites. The precise site at which methylation occurs is crucial for determining its effect on gene transcription. For instance, H3K9 methylation within the coding region of a gene has been found to correlate with transcription activation, whereas the same modification is associated with inactive transcription when found in the promoter region [39].

#### 2.2.4. Phosphorylation

Histone phosphorylation, which occurs on serine, threonine, and tyrosine residues, is most commonly associated with transcriptional activation as the negative charge of the phosphate group creates a repulsive force between the histone and the negatively charged DNA [32]. Histone phosphorylation is regulated by protein kinases (PKs) and protein phosphatases (PPs).

#### 2.2.5. Ubiquitination

In addition to acetylation, methylation, and phosphorylation, histones may be modified through ubiquitination. Histone ubiquitination refers to the covalent addition of the 76-amino-acid protein ubiquitin to histones H2A and H2B. Similar to acetylation and phosphorylation, histone ubiquitination is a reversible modification. Several studies have demonstrated that histone ubiquitination and methylation often cross-talk and act in combination as well as sequentially to regulate transcription. H2B ubiquitination is considered a prerequisite for H3 methylation, but H2A inhibits H3 methylation of the gene on which they are located [40,41].

#### 2.2.6. SUMOylation

Similar to ubiquitination, histone SUMOylation refers to enzymatic conjugation of the 100-amino-acid small ubiquitin-related modifier (SUMO) groups at specific lysine residues of histones [42]. Histone SUMOylation mediates transcriptional repression via interaction with other repressor factors and prevention of histone acetylation [43].

## 3. Smoking Modulates the Epigenome during Carcinogenesis

Cigarette smoke condensate (CSC) induces cancer-associated epigenomic alterations in cultured respiratory epithelial cells [44]. Cigarette smoke induced DNA damage occurs in nuclear and mitochondrial DNA during the early phase of lung cancer carcinogenesis [45]. Both tobacco-induced DNA changes and tobacco-induced epigenetic changes affect molecular regulatory mechanisms, including (1) receptors, (2) cell cycle regulators, (3) signaling pathways, (4) apoptosis mediators, (5) angiogenic factors, and (6) invasive and metastasis mediators, to induce carcinogenesis [46]. Here, we will focus on how smoking modulates the epigenome during carcinogenesis.

### 3.1. Smoking Induces DNA Methylation-Associated Enzymes Activity

Smoking, which is the largest preventable cause of morbidity and mortality in the world, causes various cancers such as those of the mouth and throat, voice box, esophagus, lung, stomach, kidney, pancreas, liver, bladder, cervix, colon, and rectum, as well as leukemia. In 2004, the International Agency for Research on Cancer (IARC) reported that 4000 compounds are present in mainstream tobacco smoke; among these, 200 are poisonous, and 60 carcinogens. The composition of mainstream smoke, sidestream smoke, and secondhand smoke, in terms of the content of these compounds, is similar [47]. The carcinogens present in tobacco smoke include polycyclic aromatic hydrocarbons, *N*-nitrosamines, aromatic amines, aldehydes, miscellaneous organics, and inorganic compounds. Inhalation and metabolic activation of these carcinogens during exposure to tobacco smoke often causes changes in DNA sequences, such as formation of DNA adducts [48], induction of double-stranded DNA (dsDNA) breaks [49], and development of point mutations [50,51]. Furthermore, tobacco smoke also commonly induces changes in the activity of chromatin-modifying enzymes, such as DNMTs [28,52], HATs [53], HMTs [44], histone kinases, and ubiquitinases [54], that result in epigenetic changes and abnormal cell physiology.

### 3.2. DNA Methylation in Smoking-Related Cancers

Both current tobacco use and prenatal smoke exposure strongly induce DNA methylation [28]. In the context of prenatal exposure, cigarette smoke induces hypoxia in the embryo, which in turn modulates methyl group availability [28]. Current smokers with gene-specific DNA hyper- or hypo-methylation may be highly susceptible to disease development based on their genomic DNA methylation profiles. In lung cancer, the retinoic acid receptor beta (RAR-β) has been found to be absent in varying degrees in all malignant tumor types. Tobacco smoke may play a potential role in *RAR-β* gene methylation during early pathogenesis of lung cancer [55]. Several CpG loci in *WWTR1*, *NHP2L1*, *PLA2G6*, *NFIX*, and *SMUG1*, with significant association with smoking, localize to a CpG island within the transcription start site (TSS), exon, or 5′ untranslated region (UTR) [56]. Methylation at cg05575921, a CpG residue in the aryl hydrocarbon receptor repressor (*AHRR*), is the most sensitive indicator of smoking status at all levels of smoking. Reversion of *AHRR* demethylation is a quantitative biomarker of smoking cessation, and *AHRR* methylation status is a quantifiable biomarker for lung cancer progress in smoking cessation [57]. In bladder cancer, there is a relationship between epigenetic silencing of the tumor-suppressor genes *p16* (*INK4A*), *RASSF1A*, *PRSS3*, and the four *SFRP* genes and exposure to both tobacco and arsenic. Promoter methylation-mediated silencing of each of these genes occurs in approximately 30–50% of bladder cancers. Epigenetic silencing of *RASSF1A*, *PRSS3*, or any of the *SFRP* genes is significantly associated with advanced tumor stage (*P* < 0.001, *P* < 0.04, and *P* < 0.005, respectively) [58]. Promoter methylation of *p16* (*INK4A*), *RASSF1A*, and *PRSS3* occurs in approximately 30% of cases of bladder cancer; promoter methylation of both *RASSF1A* and *PRSS3* is associated with advanced tumor stage. Arsenic exposure, measured by determination of toenail arsenic concentration, was associated with promoter methylation of *RASSF1A* and *PRSS3*, but not that of *p16INK4A* [59]. In prostate cancer, cigarette smoking-induced methylation of genes such as aldehyde oxidase 1 (*AOX1*), claudin 5 (*CLDN5*), early B-cell factor 1 (*EBF1*), homeobox A7 (*HOXA7*), lectin galactoside-binding soluble 3 (*LGALS3*), microtubule-associated protein τ (*MAPT*), protocadherin γ A (*PCDHGA*)/protocadherin γ B (*PCDHGB*), paraoxonase 3 (*PON3*), synaptonemal complex protein 2 like (*SYCP2L*), and zinc finger and SCAN domain containing 12 (*ZSCAN12*) was associated with a higher risk of recurrence and mortality [60].

### 3.3. Smoking Induces DNA Methylation

Cigarette smoke extract (CSE) treatment (0.1% CSE) induces the transformation of simian virus 40 (SV40) immortalized normal human urinary tract epithelial cells (SV40-HUC-1), and increases the methylation levels of *DCC*, *HIC1*, and *MCAM* genes [61]. Tobacco exposure, in combination with increasing age and male gender, drives and enhances the selection of promoter-hypermethylated cells in patients with bladder cancer [62]. CSE has been shown to cause hypermethylation and inactivation of the WW domain-containing oxidoreductase (*WWOX*) gene in T-24 human bladder cancer cells [63]. However, it has been shown that DNA methylation may be induced by a single compound, such as tobacco-specific nitrosamine 4-(methylnitrosamino)-1-(3-pyridyl)-1-butanone (NNK) and inorganic arsenic in CSE, acting alone. The death-associated protein (DAP)-kinase gene was inactivated in both a murine model of cigarette smoke-induced lung cancer and human non-small cell lung cancer. The DAP-kinase gene methylation rates of NNK-induced hyperplasias and adenocarcinomas were 46% and 52%, respectively [64]. High-Temperature Requirement Factor A3 (*HtrA3*) expression is reduced in lung cancer cell lines and lung tumors. NNK treatment downregulated *HtrA3* expression by increasing the methylation level of the first exon of *HtrA3* in human bronchial epithelial cells BEAS-2B and lung tumors [65]. Lysyl oxidase (LOX) propeptide is considered to act as a tumor suppressor. NNK has been shown to enhance methylation of CpG at the *LOX* promoter, but decrease histone H3 acetylation at the core promoter region of *LOX* gene in rat fetal lung fibroblasts (RFL6) [66]. Arsenic exposure was associated with promoter methylation of *RASSF1A* (*P* < 0.02) and *PRSS3* (*P* < 0.1), but not of *p16 (INK4A)* or *SFRP*, in human bladder tumor samples adjusted for stage and other risk factors. Cigarette smoking was associated with a greater than two-fold increased risk of promoter methylation of *p16 (INK4A)*, with greater risk seen in patients recent exposure. Furthermore, smoking has been shown to be significantly associated with *SFRP* methylation (*P* < 0.01) [58]. Maternal cigarette smoking during pregnancy has been associated with dysregulated expression of microRNA, which affects fetal growth and development [67]. MicroRNAs and long noncoding RNAs (lncRNAs) are epigenetic regulators of stem cell pluripotency, differentiation, and malignancy. MicroRNA-487b (miR-487b) is a tumor suppressor microRNA that is silenced by epigenetic mechanisms during tobacco-induced pulmonary carcinogenesis [68]. Cigarette smoke mediates the epigenetic repression of *miR-217* and *miR-218* during esophageal adenocarcinogenesis and transformation of human bronchial epithelial (HBE) cells, respectively [69,70]. CSE has also been shown to induce Hox transcript antisense intergenic RNA (HOTAIR) during the transformation of HBE cells [71]. Both the epigenetic silencing of *miR-218* and HOTAIR-activated epigenetic silencing of *p21* are mediated via EZH2-mediated histone H3 lysine 27 tri-methylation (H3K27) trimethylation, which is in turn induced by CSE treatment.

### 3.4. Smoking Induces Histone Modification

Cigarette smoke induces posttranslational histone modifications in specific lysine and arginine residues of histones H3 and H4 in lung cells [72]. In the C57BL/6J mouse model and in human lungs, cigarette smoke exposure elicits a significant increase in the activity of chromatin modification enzymes to inhibit *Dnmt1*, *Dnmt3a*, *Dnmt3b*, *Hdac2*, *Hdac4*, *Hat1*, *Prmt1*, and *Aurkb* expression. These genes were identified by acetylation of histone H3, at lysine 56 residue (H3K56), and of H4 at lysine 12 (H4K12) [54]. Acrolein (Acr) is a potential major carcinogen implicated in smoking-related lung cancer. Acr preferentially binds free histones rather than nucleosomal histones, and inhibits the acetylation of N-terminal tails of histones H3 and H4 to regulate nuclear import and chromatin assembly [73]. Cigarette sidestream smoke (CSS) treatment markedly induced histone H3 phosphorylation at serine 10 and 28 residues (H3S10 and H3S28) in human pulmonary epithelial cell and normal human lung fibroblasts. The phosphorylation of H3S10 was mediated by c-jun N-terminal kinase (JNK) and phosphoinositide 3-kinase (PI3K)/Akt pathways. H3S10 phosphorylation was found to occur at high levels in the promoter sites of the proto-oncogenes *c-fos* and *c-jun* [74].

## 4. Alcohol Use Known to Induce DNA Methylation during Carcinogenesis

Alcohol consumption is associated with a large number of health disorders, chronic diseases, and deaths worldwide [75]. According to the IARC Monographs, “alcohol consumption is carcinogenic to humans (Group 1); ethanol in alcoholic beverages is carcinogenic to humans (Group 1); acetaldehyde associated with the consumption of alcoholic beverages is carcinogenic to humans (Group 1)” [75]. Alcohol consumption causes cancers of the oral cavity, pharynx, larynx, esophagus, breast, colorectum, liver, pancreas and colorectum; alcohol consumption is associated with an approximately 10% increased risk of breast cancer with each drink per day [76]. Recent studies show that alcohol elicits epigenetic changes, such as DNA methylation, histone acetylation, and histone methylation, that are implicated in carcinogenesis [77]. Alcohol has additionally been shown to interfere with the epigenetic regulation of gene expression: Mahnke et al. have shown that chronic low-level drinking results in DNA hypomethylation, while high levels of drinking result in hypermethylation [76]. Acute alcohol exposure associated with the opening of chromatin as a result of increased histone acetylation and HDAC inhibition [78]. Bohacek et al. reported that moderate and heavy drinkers show subtle reductions in DNA methylation of the *H19* imprinted gene in their sperm compared with non-drinkers [79]. Changes in acetylation commonly occur at H3K9 and are regulated in specific addiction-related brain circuitry [80]. The effect of acute and chronic alcohol exposure on DNA methylation of specific promoter sequences in animal models has been investigated [81]. Previous studies have shown that DNA hypermethylation of specific genes is related to oral cancers [82]. Furthermore, Pattani et al. reported that the severity of oral cancer histopathology increases with the degree of tumor suppressor gene promoter hypermethylation [83]. Therefore, alcohol plays an important role in DNA methylation events and histone modifications that promote carcinogenesis. Future studies should focus on identifying drugs that target the epigenome, including those that modulate DNA methylation and histone modification.

## 5. Betel Quid Chewing Modulates the Epigenome during Carcinogenesis

Betel quid preparation methods vary by country. In India, betel quid comprises a mixture of areca nut, lime, tobacco, or piper betel leaf [84]. In Taiwan and Papua New Guinea, piper betel inflorescence is used instead of tobacco [85,86]. An increasing number of studies show that betel quid chewing affects the epigenetic modulation of gene expression, resulting in carcinogenesis. However, the complexity of betal quid components makes the assessment of its carcinogenic potency challenging.

The absence of *O*^6^-methylguanine-DNA methyltransferase (*MGMT*) expression [87], *CDKN2A* promoter hypermethylation [88], and *p14*, *p15*, *p16* promoter hypermethylation [89] has been detected in tumors from betel-quid chewing patients. The most common betel-quid chewing-related cancers are oral cancer and esophageal cancer. Histone methylation- and acetylation-related enzymes were found to be expressed at high levels in tumor tissue from patients with esophageal squamous cell carcinoma [90]. The histone modification-related proteins, ARK2, G9a, EZH2, and SUV39H1 are associated with poor prognosis of the male oral squamous cell carcinoma (OSCC) population in Taiwan [91]. Over 80% of cases of OSCC and over 50% of epithelial dysplasia have been reported to show positive HDAC2 nuclear staining, which is related to larger tumor size, lymph node metastasis, and shorter overall survival [92]. Betel quid chewing releases toxic chemicals into blood circulation; animal studies have shown that 3-(Methylnitrosamino) propionitrile (MNPN), which is found in the saliva of betel quid chewers, acts a potent carcinogen by promoting DNA methylation in the nasal mucosa, liver, and esophagus [93]. The predominant alkaloid and most potent carcinogen present in betel quid is arecoline [94,95,96]; this compound has been shown to alter the expression of several genes involved in histone methylation (*MII*, *Setdb1*, and *Suv39h2*), acetylation (*Atf2*), and demethylation (*JMJD6*). Further, arecoline reduces the levels of H3K9 methylation, which is involved in the stability of chromatin structure in the K-562 bone marrow cell line [97]. In animal models, treatment with a combination of 4-nitroquinoline-1-oxide (4-NQO) and arecoline has been shown to induce hypermethylation of *PARβ*, whose expression is lost during carcinogenesis [98].

## 6. Therapeutic Drugs Targeting the Epigenome

An increasing number of studies have described the association between specific lifestyle choices (smoking, betel quid chewing, and alcohol consumption) and epigenetic modulation of carcinogenesis. Numerous drugs targeting the epigenome, including those that modulate DNA methylation and histone modification, have been approved by the US Food and Drug Administration (FDA) and used for cancer therapy. According to The Human Epigenetic Drug Database (HEDD) [99], epigenetic drugs that modulate DNA methylation and histone modification are categorized into seven main types: DNA methyltransferase inhibitors (DNMTis), histone deacetylase inhibitors (HDACis), histone demethylase inhibitors (HDMis), histone methyltransferase inhibitor (HMTis), histone acetyltransferases inhibitors (HATis), inhibitor of protein binding to acetylated histone (PAHis), and inhibitor of protein binding to methylated histone (PMHis). FDA-approved human epigenetic drugs are mainly classified into two types, DNMTis and HDACis; these are listed in Table 1. The FDA-approved DNMTis and HDACis were nowadays used in the specific disease, and the anti-cancer potency is testing and evaluating in preclinical or phase 1 to 2 studies. The FDA-approved DNMTis 5-Aza-2′-deoxycytidine and 5-Azacytidine are used to treat myelodysplastic syndromes. Their potential for the treatment of cancers, including leukemia [100,101], breast cancer [102,103], lung cancer [104,105], and prostate cancer [106,107], is currently being tested in phase 1 clinical trials. To date, 5-Azacytidine has been studied in phase 2 clinical trials for non-small cell lung cancer. Hydralazine is used for the treatment of hypertension, and its anti-cancer potential for cervical [108] and breast cancer [109] is under evaluation in preclinical studies. The FDA-approved HDACis suberoylanilide hydroxamic acid and romidepsin are used to treat cutaneous T-cell lymphoma, and their efficacy against other types of cancers, such as urothelial carcinoma [110] and colorectal carcinoma [111], is in phase 1 or phase 2 testing. Panobinostat, which is used for the treatment of multiple myeloma, is currently under testing in preclinical studies for its efficacy against chronic myelogenous leukemia [112], epithelioid sarcoma [113], endometrial cancer [114], and prostate cancer [115]. Belinostat, which is used to treat peripheral T-cell lymphoma, is under phase 1 or phase 2 testing for the treatment of lung cancer [116] and multiple myeloma [117].

In addition to the FDA-approved epigenetic drugs discussed, numerous additional epigenetic drugs have been developed; these include natural compound derivatives or known drugs with novel epigenetic applications. Clorgiline (HDMi) is a monoamine oxidase inhibitor used for the treatment of depression. Its efficacy as an anti-cancer agent, for the treatment of bladder cancer and prostate cancer [118,119], is currently under evaluation in preclinical studies. Further, 3-deazaneplanocin A (HMTi), an anti-viral drug used for the treatment of Ebola viral infection, is also an EZH2 inhibitor; the combination treatment with retinoic acid and belinostat is an effective inducer of acute promyelocytic leukemia cell differentiation [120]. Hydrazinocurcumin (CTK7A), a HATi derived from curcumin, has been shown to reduce the growth of xenografted oral tumors in mice [121], reverse androgen-regulated gene expression in prostate cancer cells, inhibit the growth of xenografted castration–resistant prostate tumors [122], and attenuate the invasiveness of hypoxia-induced gastric cancer cells [123]. Resveratrol is a natural phenol and phytoalexin produced by fruit skin when the plant is under attack by pathogens. This compound acts as a pan-HDACi inhibitor, suppressing growth and HDAC activity in HepG2 hepatocellular carcinoma cell lines [124]. Trichostatin A is not only an antifungal antibiotic but also a HDACi, whose efficacy against lung cancer [125], cervical cancer [126], prostate cancer [127], and breast cancer [128] is currently being tested in preclinical studies.

Most developed epigenetic drugs are in pre-clinical, phase 1, or phase 2 testing for lung cancer, breast cancer, prostate cancer, and leukemia. However, investigations of applications for betel quid chewing-induced cancers, such as head and neck cancer and esophageal cancer, are lacking. There is a need for investigations into the use of epigenetic drug treatments on head and neck and esophageal cancer.

## 7. Conclusions

Numerous environmental factors such as diet, alcohol use, stress, and environmental chemicals are currently known to affect epigenetic regulation of gene expression, leading to increased rates of cancers and other diseases. The present review discusses the effects of specific lifestyle factors, namely smoking, betel quid chewing, and alcohol consumption, on the epigenome (Table 2). The prevention or reversal of these epigenetic changes is crucial; accordingly, the development of drugs targeting the epigenome represents a promising strategy in cancer therapy. The present review summarizes current understanding of the oncogenic effects of epigenetic aberrations induced by smoking, alcohol use, and betel chewing and drug discovery, thereby informing drug discovery aimed at the mitigation of these effects.

## Figures and Tables

**Table 1:** FDA-approved epigenetic drugs.

Category	Synonyms	Cas Number	Disease	FDA Approval Number
DNMTi	5-Azacytidine	320-67-2	Myelodysplastic syndromes	50794
	5-Aza-2′-deoxycytidine	2353-33-5	Myelodysplastic syndromes	21790
	Hydralazine	86-54-4	Hypertension	8303
HDACi	Suberoylanilide hydroxamic acid	149647-78-9	Cutaneous T-cell lymphoma	21991
	Panobinostat	404950-80-7	Multiple myeloma	205353
	Belinostat	414864-00-9	Peripheral T-cell lymphoma	206256
	Romidepsin	128517-07-7	Cutaneous T-cell lymphoma	22393

FDA: the US Food and Drug Administration; DNMTi: DNA methyltransferase inhibitor; HDACi: histone deacetylase inhibitor.

**Table 2:** Risk factors that induce epigenetic aberrations in various cancer types.

Risk Factor	Epigenetic Aberrations	Cancer Types	References
Smoke	DNA methylation	Lung cancer	[55,56,57,64,65,66] [68,69,70]
		Bladder cancers	[58,59]
		Prostate cancer	[60,62,63]
	Histone modification	Lung cancer	[72,73,74]
Alcohol	DNA methylation	Liver cancer	[77]
		Oral cancer	[82]
	Histone modification	Oral cancer	[77]
Betel nut	DNA methylation	Esophageal cancers	[88]
		Oral pre-cancerous lesions	[89]
	Histone modification	Esophageal squamous cell carcinoma	[90]
		Oral squamous cell carcinoma	[91]
		Oral cancer	[92]
